# Pathogen intelligence

**DOI:** 10.3389/fcimb.2014.00008

**Published:** 2014-01-31

**Authors:** Michael Steinert

**Affiliations:** Institut für Mikrobiologie, Technische Universität BraunschweigBraunschweig, Germany

**Keywords:** pathogen intelligence, information processing, cooperative behavior, heterogeneity, dormancy, biofilm, learning, memory

## Abstract

Different species inhabit different sensory worlds and thus have evolved diverse means of processing information, learning and memory. In the escalated arms race with host defense, each pathogenic bacterium not only has evolved its individual cellular sensing and behavior, but also collective sensing, interbacterial communication, distributed information processing, joint decision making, dissociative behavior, and the phenotypic and genotypic heterogeneity necessary for epidemiologic success. Moreover, pathogenic populations take advantage of dormancy strategies and rapid evolutionary speed, which allow them to save co-generated intelligent traits in a collective genomic memory. This review discusses how these mechanisms add further levels of complexity to bacterial pathogenicity and transmission, and how mining for these mechanisms could help to develop new anti-infective strategies.

## Introduction

Intelligence is a term which is difficult to define. Tentative working definitions of intelligence usually include perception, learning, memory and decision making. Intelligent processes are primarily studied in humans, other mammals and birds (Shettleworth, 2001). However, comparative studies using, for example, evolutionarily, distantly related cephalopods, which have a nervous system fundamentally different from that of vertebrates, have revealed highly interesting aspects of intelligent behavior such as spatial learning, navigational abilities, and predatory techniques (Alves et al., 2008). Investigations of other invertebrates such as the gastropod *Aplysia california* have led to fundamental insights into non-associative and associative learning, memory, and synaptic plasticity (Baxter and Byrne, 2012). A change of concept regarding animal intelligence was required with the advanced understanding of super organisms such as ant and bee colonies. Knowledge of these social insects led to the discovery of “distributed intelligence” or “collective intelligence,” in which many individuals with limited intelligence pool their resources to solve problems beyond the capabilities of individuals (Franks et al., 2002; Katsikopopulos and King, 2010). These social phenomena and decentralized, self-organized “swarm intelligence” in many other species including numerous invertebrates, unicellular eukaryotes and bacteria challenge definitions deduced from “human-like intelligence” (Nakagaki, 2001; Ben-Jacob et al., 2004; Jeanson et al., 2012; Reid et al., 2012; Shklarsh et al., 2012). Another challenging conceptual extension of the phenomenon intelligence concerns trans-generational cellular adaptations which exceed the lifespan of an individual. This has been proposed for microbes, which exhibit genetic and epigenetic adaptations to selective ecological pressures (Ben-Jacob, 2008; Veening et al., 2008). Thus, to avoid the fallacy of anthropocentric definitions of intelligence, more context-dependent views on cognitive-like abilities have been postulated. We obviously have to acknowledge that different species inhabit different sensory worlds, have evolved different kinds of intelligent processes and that these “species-specific intelligences” reflect different ecological niches. This new perspective led to research in “plant intelligence,” which has triggered ample discussions in the scientific community (Trewavas, 2003). A similar controversy can be expected from the topic of “microbial intelligence” which is currently gaining ground (Marijuan et al., 2010). Both fields share a new quality since they anticipate the existence of intelligence independent from neuronal networks. Much support for this view comes from computer science, which aims to design “artificial intelligence” using a hardware that is not of biological origin. Synthetic biology equipped with engineering-driven approaches also suggests adaptive computational aspects of microbial behavior (Goni-Moreno and Amos, 2012; Goni-Moreno et al., 2013). Together, animal, plant, and microbial intelligences seem to fit in a definition of “minimal intelligence” as goal-directed, context-dependent acquisition, storage, modification, and execution of adaptive processes that promote biological fitness. In this review we will focus on pathogen intelligence at different levels of complexity. Accordingly, we will highlight information processing on the single cell level and in microbial consortia. Moreover we will describe how genotypic and phenotypic diversity of pathogens allow dissociative behavior during infections and how bacterial networking contributes to the recalcitrance of clinically relevant biofilms. We proceed by presenting how pathogen intelligence challenges antibiotic therapy and vaccination and conclude with evolutionary mechanisms that enable pathogens to learn and to develop a collective memory.

## Pathogens and microbial intelligence

Bacterial pathogens have developed mechanisms which result in damage or death of particular hosts (Hentschel et al., 2000; Hill, 2012). In animal speciation, foraging and predator-prey relationships usually act as a positive feedback loop, which accelerate the development of highly differentiated sensory systems and adaptive behavior. Similarly, we observe an “escalated arms race” when pathogens and hosts coevolve (Hentschel et al., 2000; Steinert et al., 2000; Steinert, 2011). Accordingly, pathogen-host interactions represent fruitful models to study microbial intelligence. Since microbes lack neurons, pathogen intelligence must originate in other structures for information processing, transmission and storage.

## Information processing on the single cell level

**Figure 1:**
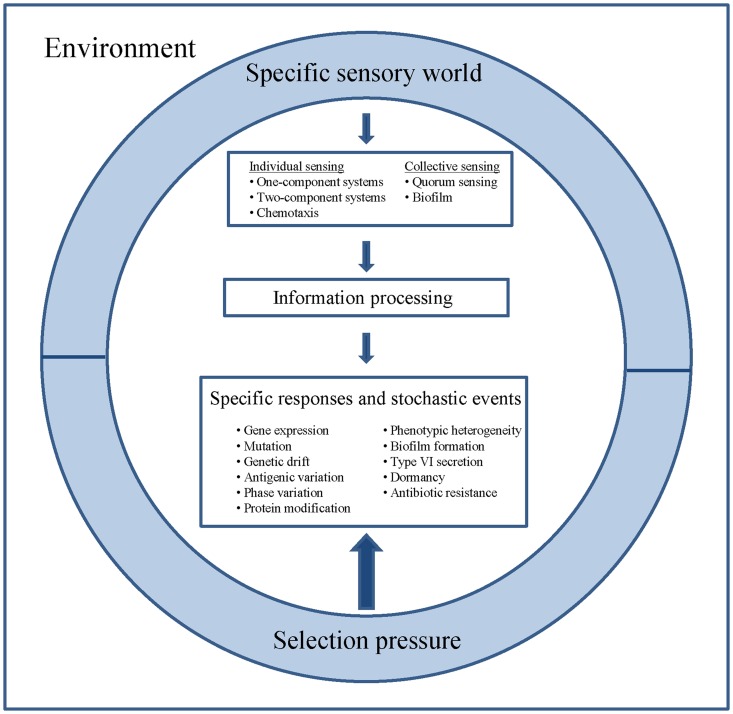
**Perception, information processing, specific responses and stochastic events of bacterial pathogens**. Different species inhabit different sensory worlds to which they respond as individual cells and as cooperative consortia. The inner- and inter species information processing of bacterial populations shapes the infectious capacity of pathogens and contributes to adaptability, division of labor, persistence and finally to epidemiologic success.

In neural networks, plasticity occurs on a variety of levels, ranging from cellular changes in neurons to large-scale alterations in neuronal anatomy and physiology (Bruel-Jungerman et al., 2007). In a parallel with the nervous system, pathogenic bacteria exhibit individual cellular sensing and behavior, as well as cooperative information processing including collective sensing, distributed information processing, joint decision making and even manipulation of the extracellular environment (Figure 1).

Bacterial cells are exquisitely sensitive to the local environment, making intricate adjustments of their behavior in adaptation to the ever-changing environment. The required information processing on the single cell level relies on stimulus-response mechanisms. This input-output scheme encouraged efforts to describe genetic switches and oscillators with mathematical models and to design functional gene circuits including logic gates and clocks. (Menolascina et al., 2012).

One- and two-component systems as well as chemotaxis play a key role in allowing bacteria to sense and respond to their environment. They are widespread in pathogenic bacteria and perceive stimuli either by a cytosolic receptor-transducer, or by a transmembrane histidine kinase that connects with a response regulator, or with an independent receptor associated to the histidine kinase, respectively (Galperin, 2005; Williams, 2007; Porter et al., 2011; Duan et al., 2013). One-component systems detect their stimuli in the cytosol and afterwards execute cellular adaptations. They appear to be the most abundant signaling strategy in pathogens. Two component regulatory systems have adapted to respond to external stimuli, including nutrients, redox state, changes in osmolarity, quorum signals, antibiotics, temperature, chemoattractants, and pH (Heithoff et al., 2012). Chemotaxis systems have incorporated additional non-kinase receptors for activating the protein kinase. If the bacterium senses a higher concentration of an attractant, it delays tumbling and sustains its straight movement. If it is moving to a repellent, it will tumble sooner and try a new direction at random. Since bacterial cells are too short to detect chemical gradients, they use temporal sensing to optimize their living conditions (Porter et al., 2011).

## Cooperative bacterial information processing

A fundamental problem in neuroscience is to understand events occurring within structured networks that contribute to learning and memory. Similarly, our knowledge on cooperative information processing in microbial communities is still rudimentary compared to our understanding of microbial competition on the cellular level. However, studies on quorum sensing, bacterial interactions through small molecules, proteins and genetic elements, electron transfer and cross-talk in microbial communities such as biofilms are changing our awareness of bacterial synergisms (Kovacic, 2007; Ng and Bassler, 2009; Juhas, 2013; Tashiro et al., 2013). Meanwhile, it is well accepted that inner- and inter-species information processing of bacterial populations shapes the infectious capacity of pathogens and contributes to adaptability, division of labor, persistence and, last but not least, to learning and memory to cope with the immune system (Figure 1).

Cooperative perception in bacterial colonies extends the sensing abilities of bacterial cells and enables them to gather data from distant surroundings. Especially quorum sensing, which describes the ability of bacteria to sense cell densities, has been shown to be critical for the establishment of infections. Quorum sensing controls features such as virulence gene regulation, antibiotic resistance, swarming motility, conjugational plasmid transfer, and biofilm maturation (Ng and Bassler, 2009).

Outer membrane vesicles (OMVs), which are shed from Gram-negative bacteria, are also means by which bacteria interact with other bacteria and eukaryotic cells (Kuehn and Kesty, 2005; Galka et al., 2008; Shevchuk et al., 2011). They can kill other bacteria by delivering toxic factors, but also they transfer DNA and antibiotic-resistance enzymes. Modulation of biofilm formation and quorum sensing functions have also been assigned to OMVs. In host tissues, OMVs seem to play a significant role in tissue destruction and inflammation (Jäger et al., 2014).

In addition to small molecules, volatile organic compounds, proteins and vesicles, horizontally transferred genetic information is involved in the dissemination of antibiotic resistance and virulence properties (Wiedenbeck and Cohan, 2011; Juhas, 2013). Effective horizontal gene transfer mechanisms such as conjugation, transformation and transduction contribute to a joint gene pool in diverse populations and thus ensure the access to a collective genetic memory. Interestingly, a recent study in the field of synthetic biology demonstrated that bacterial conjugation can be used for wiring logical functions between different cell types in engineered microbial consortia (Goni-Moreno et al., 2013).

## Genotypic and phenotypic diversity allow dissociative behavior of pathogenic consortia

**Figure 2:**
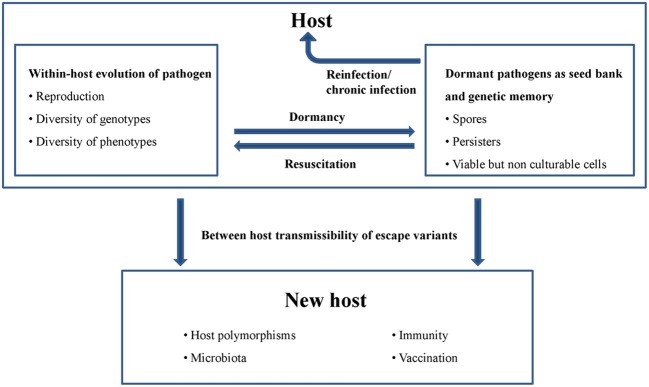
**Genotypic and phenotypic diversity of pathogens and bacterial dormancy strategies contribute to within-host evolution, chronic infections, genetic memory and between host transmissibility**.

To be successful, pathogenic microbes must resist the innate and adaptive immune system of their host and in many cases tolerate prolonged antibiotic exposure. A consequence of these selection pressures is that the pathogen type that enters a host usually differs genetically from the type that leaves the host (Brunham et al., 1993; Alizon et al., 2010). In spite of this “within-host evolution,” the resulting escape variants need to ensure their “between-host transmissibility.” These sometimes conflicting prerequisites of pathogen fitness and epidemiologic success are further exacerbated by the fact that pathogens encounter host polymorphisms. A strategy of pathogens to solve this dilemma is to maintain a high level of diversity of genotypes and phenotypes (Figure 2). Major driving forces for the generation of microbial diversity within a population are mutation, genetic drift and selection. However, some pathogens, such as e.g., *Neisseria*, *Streptococcus*, and *Mycoplasma* species, additionally store the information for various alternative forms of surface antigens in their genomes (Van der Woude and Bäumler, 2004). Antigenic variation is the capacity of a pathogen to systematically express alternative forms of particular immundominant surface antigens, whereas phase variation describes the on-off expression of particular antigens. Antigenic variation and phase variation facilitate evasion from immunologic memory and thus promote persistent infections, coexistence of serotypes in a host, and transmission. Especially vector-borne pathogens use antigenic variation to prolong their circulation in the host and thus increase the likelihood of uptake and transmission by an appropriate vector (Lange and Ferguson, 2009).

In addition to the described genetic mechanisms, phenotypic heterogeneity, and dormancy of microbes also seem be of great importance for pathogen survival and contribute to a kind of bacterial memory. Phenotypic heterogeneity is a phenomenon in which genetically identical bacterial cells acquire distinct phenotypes. The formation of isogenic subpopulations can result from epigenetic mechanisms such as amplified noise in gene expression or patterns of DNA methylation. Since these cell states can be inherited to the next generation epigenetic mechanisms constitute a kind of non-genomic memory (Ben-Jacob, 2008; Veening et al., 2008). Dormancy describes a reversible state of low metabolic activity and includes bacterial spores, persisting and viable but non-culturable cells (Steinert et al., 1997; Lewis, 2007; De Jong et al., 2011). Both strategies ensure that subpopulations are well prepared for unpredictable changes in the environment.

Phenotypic heterogeneity of isogenic populations via stochastic variations in gene expression can be viewed as a risk-spreading strategy and has the potential to increase the overall fitness of the species. The analysis of genetically homogenous populations under rapidly changing conditions, artificial gene networks and mathematical modeling revealed that positive or double-negative feedback loops in regulatory networks provide efficient switching mechanisms for the generation of bistable or multistable outputs at the population level. “Bimodal decision making” is involved in adaptive processes such as sporulation, control of virulence genes, biofilm formation and the activation of horizontal gene transfer and exhibits some degree of hysteresis. This common feature of bistability describes the dependency of the steady-state response curve on the direction of the parameter change, which may be viewed as a kind of memory-like characteristic (Smits et al., 2006; Mitrophanov and Groisman, 2008; Veening et al., 2008; Turner et al., 2009).

A pre-existing heterogeneity of distinct physiologies confers selective advantages and may be faster than sensing and responding under harsh selection pressures like antibiotic treatment. Moreover it generates subpopulations which have distinct functions during pathogenesis. For *Salmonella typhimurium*, e.g., it was demonstrated that inflammatory and non-inflammatory subpopulation together accelerate systemic spread within the host (Stewart and Cookson, 2012).

The surmounting of adverse host conditions and the establishment of chronic infections are also often achieved by bacterial dormancy, including slow growing persister cells or sporulation. These cellular specializations of dormancy seem to proceed through stochastically and reversible steps toward irreversible, all-or-none decisions (Lewis, 2010). The resulting bacterial stages exhibit an extraordinary resistance. They represent a reservoir for perpetual re-infections and may buy evolutionary time for the pathogen population to develop additional survival mechanisms against harsh immune responses or antibiotic treatments.

Interestingly, repeated transitions to and from dormancy may establish a seed bank which avoids a genomic bottleneck situation and helps to maintain or generate a high level of microbial diversity (Jones and Lennon, 2010). Thus, dormancy may prevent genotypes which are only successful under certain conditions from outcompeting others, a factor which may be relevant at later stages of infection. Since the selection pressure during the pathogen-host interaction changes and favors different phenotypes at different times, dormancy seems to be an integral part of labor division and genetic memory in microbes. This survival strategy allows pathogens to deploy suitable cells in response to, and in anticipation of, various adverse conditions.

## Interplay of anatomical structure and functional activities in biofilms

From the discussion above, it is evident that there is a tremendous amount of interaction going on at all scales of microbial consortia. Interbacterial communication, collective and dissociative behavior, cooperative decision-making, cellular division of labor and differentiation processes are particularly advantageous attributes of habitation in microbial biofilms. They play an important role in infectious diseases such as cystic fibrosis, peridontitis, urinary tract infections and native valve endocarditis (Donlan and Costerton, 2002). Analagous to neural network dynamics, spatiotemporal biofilm dynamics exhibit a reciprocal interplay between anatomical structure and functional activities (Serra et al., 2013).

The striking morphological differences between the surface and basis indicate a pronounced stratification of biofilms. Accordingly, it is widely accepted that biofilms contain numerous different zones that are physiologically distinct (Donlan and Costerton, 2002; Häussler, 2010; Serra et al., 2013). The resulting microenvironments in a biofilm induce specific developmental programs, which lead to the expression of properties not predictable from those of individual bacterial cells. Thus, high cell densities, concentration gradients, structural heterogeneity, enhanced genetic diversity e.g., by generation of an endogenous oxidative stress or increase of competence under colonial stress, add further levels of complexity to bacterial adaptability in this multicellular setting.

Overall, biofilms have great relevance in microbial pathogenesis. The establishment of chronic infections and the surmounting of different adverse environmental conditions including antibiotic therapy, for instance, are not mainly due to the diffusion barriers against components of the immune system or antimicrobials, but rather to interbacterial communication and the generation of cellular specialization within biofilms. In this regard, it appears that persister cells are especially important for biofilm resilience (Lewis, 2010). The finding that the exit from dormancy requires intercellular signaling and “waking-up pheromones” further corroborates that biofilms represent heterogenic bacterial structures in which distributed information processing and joint decision making occur.

A widespread mechanism by which Gram-negative bacteria negotiate interactions with competitors is type VI secretion (Miyata et al., 2013). The type VI secretion system (T6SS) translocates toxic effectors into adjacent cells utilizing a contractile phage sheat-like structure (Basler et al., 2013). The regulatory mechanisms governing T6SS gene expression vary widely from species to species and in *Pseudomonas aeruginosa* it was shown that sister cells avoid inhibiting each other by encoding immunity proteins. Moreover, *P. aeruginosa* cells respond to the T6SS activity of adjacent sister cells by increasing their own T6SS activities. This allows *P. aeruginosa* to counterattack and prey upon heterologous bacterial species. Interestingly, this counterattack is highly selective since it is only directed against T6SS-positive attackers (Basler et al., 2013).

Another extreme form of cooperation, which has been identified as an important mechanism for biofilm development and natural transformation, is programmed cell death (PCD) in bacteria (Lewis, 2000; Schultz et al., 2012). Since PCD does not benefit the cells undergoing lysis, many questions concerning the cost-benefit relationship of this potentially altruistic trait of bacterial populations remain to be addressed (Strassmann et al., 2011). One approach to decode the principles of such a phenomenon is the construction of *de novo* gene circuits inside bacterial cells (You et al., 2004). The integration of a programmed cell death response into a bacterial quorum sensing system in *E. coli* resulted in a stable steady state in terms of cell density. This suggests that a selection pressure favoring a signaling network of general relevance may allow the development of a cell lysis programme as a subset.

In general PCD can occur through toxin-antitoxin modules, prophage activation and RecA-dependent apoptosis and leads to the release of public goods such as nutrients and virulence factors (Tanouchi et al., 2013). Moreover, PCD seems to be an integral part of biofilm dynamics. Interestingly, PCD mechanisms also play an important role in mammalian brain development and self-renewal (Kalinichenko and Matveeva, 2008).

## Pathogen intelligence challenges antibiotic therapy and vaccination

Humans have added antibiotic therapy and vaccination to their defense arsenal. These inventions of human intelligence have destabilized the existing host-pathogen equilibria, increased human life expectancy, and influenced pathogen evolution. The efficacy of antibiotic treatment is rapidly decreasing as a result of the continual spread of antibiotic resistance in microbial populations (Espinal et al., 2001; Fry, 2013). Vaccination, which pre-arms adaptive immune responses to eliminate pathogens at first encounter, led to a phenomenon called “vaccine-induced pathogen strain replacement” (Martcheva et al., 2012). This phenomenon is a consequence of vaccines, which change the competitive balance between dominant and subdominant strains, and allow non-prevalent variants or escape mutants of the pathogen to become dominant at the population level. Antibiotic resistance and pathogen strain replacement both weaken disease control and have exposed a critical need for new drugs that are not merely modifications of older inhibitors.

Mining the social behavior of pathogens, including cell division, metabolite-sensing riboswitches, bacterial secretion systems and quorum sensing, could reveal novel antibacterial drug targets. A better understanding of bacterial population dynamics and technological advances such as polyvalent vaccines, polysaccharide conjugation, recombinant protein expression, and new adjuvants may improve the induction of the appropriate type of immunity.

In addition to the attempt to combat microbial evolution with intelligent scientific research, it appears promising to beat pathogens at their own game. Although much eclipsed upon the advent of antibiotics, the use of phages for treatment of bacterial infections (phage therapy) may be reconsidered (Chan et al., 2013). Phages co-evolve with their bacterial hosts, which is an appealing property for a modern antibacterial agent.

## Conclusion

“Learning”, as behavioral modification to environmental challenge on the basis of experience, and “memory” as internal representation of the environment, evolved in unrelated biological lineages and by different means. Accordingly, similarities between neuronal intelligence and pathogen intelligence appear inviolable at the system level, but are not striking at the level of the components. Like neurons, bacterial pathogens act both as individual cells and by interacting in complex networks. Without centralized control, pathogens collectively colonize their hosts, utilize host resources, and evade host defenses. To tackle these tasks, which are far beyond the abilities of an individual cell, they cooperate as synergistic entities, generate genotypic and phenotypic diversity, establish spatiotemporal dynamics, develop morphologically adapted tissue-like structures and often take advantage of dormancy strategies. High evolutionary speed allows successful co-generated intelligent traits to be saved in a collective genomic memory. Moreover, trans-generational epigenetic mechanisms generate inheritable phenotypic diversity which contributes to a non-genomic memory.

In summary: Learning and memory in neuronal networks involve improved responses to stimuli as well as error-assessment and have evolved to optimize fitness. To what extent the underlying processes rely on repeated, varied attempts, which are continued until successful communication of neural cells is established, remains an open question in neurobiology. Pathogenic consortia obviously use this trial-and-error approach by utilizing evolutionary mechanisms such as variation, selection, networking and maintenance of diversity. If the emerging outcome of bacterial problem solving can be called “intelligent” is rather a semantic than a scientific question.

### Conflict of interest statement

The author declares that the research was conducted in the absence of any commercial or financial relationships that could be construed as a potential conflict of interest.
